# The roles of microbial products in the development of colorectal cancer: a review

**DOI:** 10.1080/21655979.2021.1889109

**Published:** 2021-02-22

**Authors:** Yongkun Fang, Cheng Yan, Qi Zhao, Jiaming Xu, Zhuangzhuang Liu, Jin Gao, Hanjian Zhu, Zhujiang Dai, Daorong Wang, Dong Tang

**Affiliations:** a Department of Clinical Medical College, Dalian Medical University, Dalian, Liaoning, P.R. China; b Department of Clinical Medical College, Yangzhou University, Yangzhou, P.R. China; c Department of General Surgery, Institute of General Surgery, Clinical Medical College, Yangzhou University, Yangzhou, China

**Keywords:** Human gut microbiome, colorectal cancer, metabolism, short-chain fatty acids, secondary bile salts, bacterial toxin

## Abstract

A large number of microbes exist in the gut and they have the ability to process and utilize ingested food. It has been reported that their products are involved in colorectal cancer development. The molecular mechanisms which underlie the relationship between gut microbial products and CRC are still not fully understood. The role of some microbial products in CRC is particularly controversial. Elucidating the effects of gut microbiota products on CRC and their possible mechanisms is vital for CRC prevention and treatment. In this review, recent studies are examined in order to describe the contribution metabolites and toxicants which are produced by gut microbes make to CRC, primarily focusing on the involved molecular mechanisms.

**Abbreviations**: CRC: colorectal cancer; SCFAs: short chain fatty acids; HDAC: histone deacetylase; TCA cycle: tricarboxylic acid cycle; CoA: cytosolic acyl coenzyme A; SCAD: short chain acyl CoA dehydrogenase; HDAC: histone deacetylase; MiR-92a: microRNA-92a; KLF4: kruppel-like factor; PTEN: phosphatase and tensin homolog; PI3K: phosphoinositide 3-kinase; PIP2: phosphatidylinositol 4, 5-biphosphate; PIP3: phosphatidylinositol-3,4,5-triphosphate; Akt1: protein kinase B subtype α; ERK1/2: extracellular signal–regulated kinases 1/2; EMT: epithelial-to-mesenchymal transition; NEDD9: neural precursor cell expressed developmentally down-regulated9; CAS: Crk-associated substrate; JNK: c-Jun N-terminal kinase; PRMT1: protein arginine methyltransferase 1; UDCA: ursodeoxycholic acid; BA: bile acids; CA: cholic acid; CDCA: chenodeoxycholic acid; DCA: deoxycholic acid; LCA: lithocholic acid; CSCs: cancer stem cells; MHC: major histocompatibility; NF-κB: NF-kappaB; GPR: G protein-coupled receptors; ROS: reactive oxygen species; RNS: reactive nitrogen substances; BER: base excision repair; DNA: deoxyribonucleic acid; EGFR: epidermal growth factor receptor; MAPK: mitogen activated protein kinase; ERKs: extracellular signal regulated kinases; AKT: protein kinase B; PA: phosphatidic acid; TMAO: trimethylamine n-oxide; TMA: trimethylamine; FMO3: flavin-containing monooxygenase 3; H_2_S: Hydrogen sulfide; SRB: sulfate-reducing bacteria; IBDs: inflammatory bowel diseases; NSAID: non-steroidal anti-inflammatory drugs; BFT: fragile bacteroides toxin; ETBF: enterotoxigenic fragile bacteroides; E-cadherin: extracellular domain of intercellular adhesive protein; CEC: colonic epithelial cells; SMOX: spermine oxidase; SMO: smoothened; Stat3: signal transducer and activator of transcription 3; Th17: T helper cell 17; IL17: interleukin 17; AA: amino acid; TCF: transcription factor; CDT: cytolethal distending toxin; PD-L1: programmed cell death 1 ligand 1

## 1. Introduction

Colorectal cancer (CRC) is the world’s third most common malignancy and second leading cause of cancer death [1]. Although extensive research has been conducted, the exact cause and etiopathogenesis of CRC are yet to be fully clarified. Due to technological advancements, such as high-throughput sequencing, changes in human gut microbiome type and abundance in CRC patients have been identified [2,3]. There are approximately 38 trillion bacteria in the human body, and the intestine is the organ which is most densely colonized [4]. The colon also contains a minimum of two orders of magnitude more bacteria than any other organ [5]. The human gut microbiome is a complex community that is composed of bacteria, archaea, viruses, and eukaryotes [6]. This complex ecosystem contains approximately 500 different bacteria species [7]. These intestinal bacteria are mainly composed of *bacteroidetes, firmicutes, actinomycetes, proteobacteria*, and *ruminococcaceae* in addition to relatively few *clostridium* [8]. As well as the regulation of immunity and maintenance of human health, the human gut microbiome also mediates the occurrence and development of some diseases, which includes CRC [9,10].

It is commonly believed that intestinal microbiota products, including butyrate, H_2_S, and bacterial toxins, contribute to CRC’s development and progression [11–13]. Butyrate, for example, displays significant wellness promoting and anti-tumor characteristics. It is the prime energy resource for colon cells, maintaining epithelial integrity and inhibiting inflammation and cancer by the role it plays in immunity, gene expression, and epigenetic regulation [13]. However, the molecular mechanisms which underlie the effects of gut microbial products on CRC have not yet been fully clarified, and the effect of some products on CRC is still controversial. Extensive studies have explored the relationship between gut microbes, their products, and CRC, and their relationship will be explored based on recent studies. This review examines the contribution some products of the human gut microbiome have made to the development of CRC, in particular the molecular mechanisms between products and CRC, to explore methods of preventing and treating CRC using these controllable factors.

## 2. Factors inhibiting colorectal cancer

### 2.1. Butyrate inhibits the invasion and proliferation of CRC and promotes the apoptosis of cancer cells

Colon bacteria break down indigested dietary fibers and starches and produce short-chain fatty acids (SCFA), such as acetic acid, propionic acid, and butyric acid [14]. The aerobic glycolysis of SCFA provides colon cells with their main energy source [15], while also playing a part in the immunity and metabolism of the host intestine. SCFA content in CRC patients’ plasma decreases significantly, which proves that a decrease in SCFA promotes CRC progression [16].

Butyrate is the most studied short-chain fatty acid and it is mostly synthesized by glycolysis from hydrocarbons by two families of the firmicutes of the human colon, ruminococcaceae and lachnospiraceae [17]. As a histone deacetylase (HDAC) inhibitor, butyrate inhibits carcinoma cell multiplication while triggering cell death [18]. In normal colon cells, butyrate is β-oxidized by mitochondria and produces energy from the tricarboxylic acid cycle (TCA cycle) or cytosolic acyl coenzyme A(CoA). Otto Warburg et al. remarked that carcinoma cells have the ability to alter their metabolic modes even under oxygen, and they prefer to undergo a glycolytic pathway rather than an oxidative phosphorylation (OXPHOS) pathway, in order to transform the absorbing glucose mostly into lactate [19]. The transformation of glycolytic metabolism has been recognized as being a dominant characteristic of carcinoma cells; cancerous colon cells prefer glucose to butyrate as their preferred energy resource due to the Warburg effect pathway. As a result, cancerous colon cells accumulate a large amount of butyrate which acts as HDAC inhibitor [20]. Butyrate can enter the nucleus directly and inhibit histone deacetylase 1 and cause a reduction in short-chain acyl CoA dehydrogenase (SCAD) levels, which is the primary process in the catalyzation of mitochondrial butyrate oxidation [21]. This reduces the auto-oxidation of butyrate in CRC cells [22] and allows butyrate to accumulate in carcinoma cells, thereby restraining CRC development. This also explains why tumor cells have a greater sensitivity to histone deacetylase (HDAC) inhibitors than non-transformed cells [23].

The overexpression of microRNA-92a (MiR-92a) in CRC [24] facilitates CRC growth and invasion through the targeting of kruppel-like factor 4(KLF4) and downstream p21 [25], and a reduction in miR-92a can cause apoptosis of cancer cells [26]. MiR-92a also inhibits phosphatase and tensin homolog (PTEN) expression [24], which is a typical anti-oncogene that can be found in region 10q23 of chromosome 10 and is the foremost negative regulator of the phosphoinositide 3-kinase(PI3K) signaling pathway [27]. When faced with extracellular stimuli (including insulin, growth factors, and chemokines), activated PI3K converts PIP2 (phosphatidylinositol-4,5-bisphosphate) into PIP3 (phosphatidylinositol-3,4,5-trisphosphate) and this phosphorylates and activates Akt (protein kinase B). PTEN antagonizes PI3K by dephosphorylating PIP3 and forming PIP2 (thereby blocking the PI3K signaling cascade) [28]. Butyrate can down-regulate miR-92a expression via c-Myc, which reduces the proliferation of colon cancer cells and stimulating apoptosis [29]. Butyrate can also reduce the phosphorylation of Akt1 (protein kinase B subtype α) and ERK1/2 (Extracellular signal–regulated kinases 1/2) by blocking HDAC3 activity and inhibiting any subsequent cell movement, which ultimately impedes CRC cell metastasis and invasion [30].

**Figure 1.**
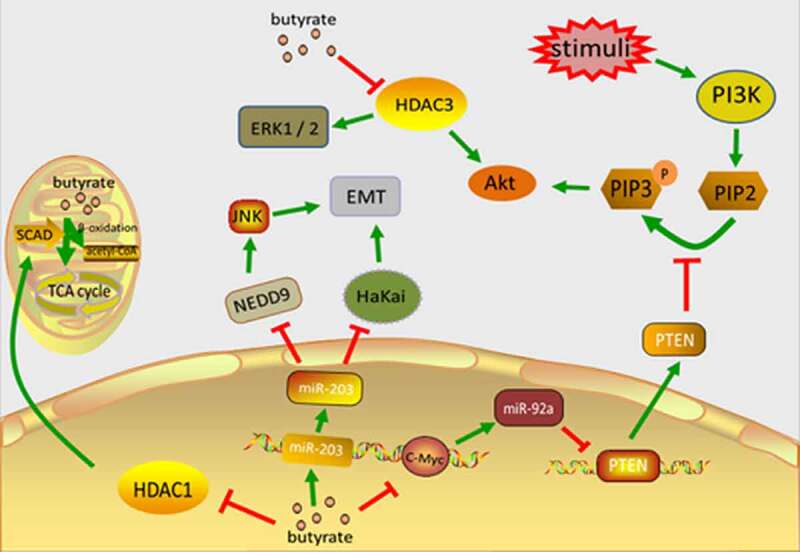
The main mechanism of butyrate inhibiting the occurrence and development of CRC. Butyrate directly enters the cell nucleus to inhibit HDAC1, reduces SCAD level, and reduces the self-oxidation of butyrate in carcinoma cells. Butyrate accumulates in cancer cells and inhibits their proliferation. Butyrate can block the activation of HDAC3, leading to decreased phosphorylation of Akt1 and erk1/2, thereby inhibiting cell motility and ultimately CRC cell migration and invasion. Butyric acid regulates the expression of c-Myc, inhibits the transcription of miR-92a, increases the expression of PTEN, and therefore antagonizes the effect of PI3K, thereby reducing the proliferation of colon cancer cells and stimulating apoptosis. Butyrate upregulates miR-203 which directly targets HaKai, reducing its level and inhibiting cell proliferation

MiR-203 expression levels are significantly reduced in CRC tissues and carcinoma cell lines, and this low expression relates to tumor size and pathologic staging (pTNM) [31]. Previous studies have demonstrated that in the early progression of cancer, epithelial cells are subjected to a procedure known as epithelial-mesenchymal transition (EMT). This is evidenced by the absence of E-calcium adhesion protein (the main ingredient of adhesion) and results in interrupted cell-to-cell contact. Hakai is an E3 ubiquitin ligase which binds and degrades E-cadherin in a phosphorylation-dependent way, regulating cell adhesion. Hakai expression is upregulated in colorectal adenocarcinomas and adenomas using differentiated TNM staging (I–IV) and compared with healthy human colon tissue. Hakai overexpression in epithelial cells also induces cell transformation, mesenchymal and invasive phenotypes, while inhibiting E-cadherin promotes increases proliferation and the oncogenic potential of the N-cadherin expression [32]. MiR-203 directly targets Hakai and lowers its level, thereby inhibiting cell proliferation. NEDD9 (neural precursor cell expressed developmentally down-regulated9), also known as HEF1 or cas-1, is a part of the crk-associated substrate (CAS) family and has a high level of expression in multiple carcinoma types, involving the adherence, migration, and invasion of cancer cells. NEDD9 promotes EMT in CRC through the JNK (c-Jun N-terminal kinase) pathway [33]. MiR-203 targets NEDD9 in order to downregulate, thereby inhibiting CRC cell multiplication, colonization, and invasion, and inducing apoptosis in CRC cells [34]. In addition to inhibiting tumor development and metastasis, miR-203 also inhibits CRC’s chemical resistance [35]. Butyrate upregulates miR-203, which inhibits CRC cell multiplication, colony formation, and invasion, and promotes CRC cell apoptosis [34](Figure 1).

The bacterium *Propionibacterium*, which is found in fibrous foods and dairy products, creates SCFAs, mainly propionates and acetate. Acetate inhibits CRC cell multiplication and triggers CRC cell apoptosis in a dose-dependent manner. However, the precise mechanism by which it transports across the CRC cell membrane is not completely understood [36,37]. Studies suggest that monocarboxylate transporter-1 (MCT1) and aquaporins play crucial roles in acetate uptake [38]. Acetate has the ability to trigger apoptosis and inhibit cell proliferation in CRC cells by DNA fragmentation and caspase-3 activation. However, acetate cam also induce the release of histone D into the cytoplasm, thereby protecting CRC cells from acetate induced apoptosis through the degradation of damaged mitochondria [39,40]. It is believed that propionate prevents colorectal cancer, but a lack of studies of its mechanism have been conducted. It is suggested by evidence that the protein arginine methyltransferase 1 (PRMT1) is overexpressed in early CRC and its high expression has an association with poor CRC patient prognosis. Recent studies suggest propionate induces PRMT1 downregulation and therefore apoptosis in CRC cells. Unfortunately, the exact mechanism which connects propionate and PRMT1 regulation remains unclear [41]. Further studies are required for the investigation of whether acetate and propionate protect against CRC and the magnitude of their inhibitory effect on CRC, particularly the mechanisms of how they are involved in CRC development.

### 2.2. Ursodeoxycholic acid (UDCA) inhibits CRC by modulating inflammatory responses and enhancing immune surveillance

Bile acids (BAs) are made in the liver from cholesterol and are then transported into the intestine by bile which promotes the intestine’s uptake of fat. Two main BAs, cholic acid (CA) and chenodeoxycholic acid (CDCA), are produced in the human liver by the ‘classical’ pathway [42]. More than 90% of intestinal BAs are reabsorbed in the ileum and then transported through the portal vein to the liver, where they are processed by hepatocytes and secreted again into the bile [43]. Bacteria in the gut, including *Clostridium, Enterococcus, Bifidobacterium*, and *Lactobacillus*, convert unabsorbed BAs into hydrophobic secondary bile salts. For example, gut bacteria transform CA into deoxycholic acid (DCA), and CDCA into lithocholic acid (LCA) [44–46].

UDCA is a secondary bile acid which is produced by *Clostridium* species, including *Clostridium absonum*, and *Clostridium baratii*. It has a chemical structure that is quite similar to that of DCA, but unlike the hydrophobic bile acid DCA, UDCA has been proven to impede colon cancer occurrence [47,48]. Patients with colorectal adenoma who have taken UDCA for a long period of time are less likely to relapse following the removal of the colorectal adenoma, and the proliferation of colonic epithelium is significantly reduced [49]. UDCA can also significantly reduce atypical adenoma’s recurrence rate [50]. UDCA can inhibit CRC in several ways, including by increasing the hydrophilicity of the bile pool, decreasing the concentration of hydrophobic BA [51], and regulating oxidative stress in colon cancer cells and colon cancer stem cells (CSC) [52]. Furthermore, UDCA up-regulates colonic major histocompatibility(MHC) expression, which enhances the immune surveillance of tumors [53], suppresses cox-2 in CRC [47], and inhibits NF-kappaB (NF-κB) activated IL-1 and deoxycholic acid induced Aβ and AP-1 in human CRC cells [54]. However, some studies have suggested that UDCA does not have a preventive effect on CRC [55]. In addition, high doses of oral UDCA are linked to a higher risk of CRC [56]. The impact UDCA has on CRC is still controversial and further studies are required in order to prove its function.

### 2.3. Niacin acts on G protein-coupled receptors (GRA) and prostaglandin receptors to inhibit colonic inflammation and carcinogenesis

In addition to being obtained from food, vitamin B is produced by the intestinal microbiota, for example, *Lactobacillus acidophilus*. Niacin, which is also called nicotinic acid or vitamin B3, acts as a precursor to coenzymes, including nicotinamide adenine dinucleotide (NAD) and nicotinamide adenine dinucleotide phosphate (NADP), and its presence is indispensable for viable cells [57]. In addition to its hypolipidemic effects, it is believed that niacin has anti-inflammatory effects [58]. Niacin signals through GPR109a, and GPR109a signaling enhances the anti-inflammatory effects of colonic macrophages and dendritic cells, allowing them to induce Treg cell and IL-10-producing T cell differentiation. Animal studies suggest that niacin can prevent colitis and colon cancer in mice through the activation of GPR109a, although the exact molecular mechanism of this remains unclear [59]. Some experiments have suggested that niacin can protect the intestinal mucosa by reducing the level of TNF-α through GPR109a [60]; whereas others have implied that niacin achieves its protective effect on the intestinal mucosa through the mediation of the release of prostaglandin D2 through GPR109a [61]. CSC intervene in tumor development and sustainment; the cells are chemically resistant and characterized by self-replenishing, multipotency, flexibility, and diversification. The elimination of CSC may increase patient survival rate [62]. Niacin has also shown effects on tumor stem cells, with small doses favoring cell proliferation in colon CSCs, and high doses inducing cell death [63]. However, as of yet, no studies have been conducted on the mechanism of this phenomenon. Many gaps remain regarding whether niacin can prevent CRC and the way in which it protects the intestinal mucosa from inflammation and CRC.

## 3. Factors promoting colorectal cancer

### 3.1. Different secondary BAs have different effects on CRC by causing oxidative stress, activating MAPK cascade, and other mechanisms

Those who follow high-fat diets generate a greater amount of secondary BA, mostly DCA and LCA, and have a higher incidence of CRC [64,65]. Cholesterol is a component of the lipid membrane which is essential and causes hardening of the membranes [66]. The secondary BA is a cholesterol derivative with washing characteristics, so when they are present in high levels, it is possible that they cause the destruction of cell membranes and local disruption to the intestinal epithelium [67]. This injury then stimulates the repair mechanism that is involved in the inflammatory response and the over-proliferation of undifferentiated cells. The over-proliferation of colonic mucosa is considered as being one of the initial steps in CRC development. Additionally, serum DCA levels have been found to be correlated with the rate of hyperplasia of the colonic mucosa [68]. Hydrophobic bile acids can produce reactive oxygen species (ROS) and reactive nitrogen substances (RNS), which cause oxidative stress, damage to DNA and proteins, and destruction of the base excision repair (BER) pathway [69]. The BER pathway can address DNA oxidation injuries facilitated by ROS. DNA repairs defects that are caused by oxidative damage as a CRC risk factor [70]. BA can also cause genomic instability via the oxidative damage pathway [67]. In the carcinogenic methane peroxide-induced rat tumor model system, DCA has been proven to raise the rate of CRC, and tumors in K-ras point mutual mutations [70].

**Figure 2.**
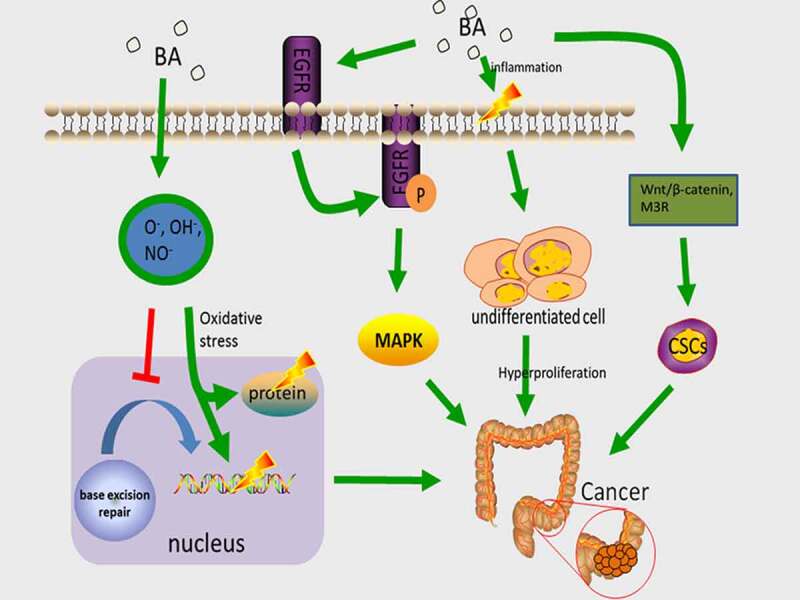
The main mechanism of BA carcinogenesis. Hydrophobic bile acids produce ROS and RNS, damage DNA and proteins, and damage BER, increasing the incidence of mutations. DCA induces PA co-localization with ERGF, promoting EGFR dimerization/multimerization and activating the MAPK cascade. activation of MAPK triggers colonic mucosal hyperproliferation, causing the development of colorectal tumors. Bile acids regulate M3R and Wnt/beta-catenin signaling and induce CSC in colonic epithelial cells, thereby inducing colon carcinogenesis

Epidermal growth factor receptor (EGFR) is a tyrosine kinase receptor, an ErbB family protein which promotes proliferation [67], invasion, or metastasis of various tumors, including CRC, by mutation or overexpression [71,72]. Through ligand stimulation, EGFR is dimerized, and the dimerization of EGFR is followed by receptor internalization and autophosphorylation, serving as the binding site for recruiting signal transducers and intracellular signal transduction cascade activators. EGFR-linked activation of the mitogen-activated protein kinase (MAPK) cascade facilitates the regulation of downstream molecules ERKs and Akt [73]. Of all the diverse subfamilies of the MAPK pathway, ERK1/2 promote cells differentiation, division, and block apoptosis, whereas p38 MAPK and SAPK/JNK1/2 induce apoptosis. Therefore, the balance of these pathways’ dynamics is a key factor for the determination of cell fate and processes. Abnormal activation of MAPK triggers colorectal mucosal overgrowth, which leads to the formation of colorectal tumors [74]. Phosphatidic acid (PA) is one of the crucial components of EGFR signaling nanodomains on cell surfaces. Secondary bile acid DCA significantly enhances the local spatial aggregation of phospholipid acid and induces co-localization between PA and EGFR, which promotes EGFR dimerization/oligomerization, and stimulates EGFR-MARK signaling [75]. DCA stimulates cell proliferation in addition to inducing EGFR phosphorylation in ligand-dependent manner [76], meaning that the participation of natural ligand (EGF) is required for EGFR activation by DCA. However, it has also been discovered that DCA regulates MAPK activation through calcium signaling [77]. In addition, BA, particularly DCA and LCA, trigger colon cancer development through the regulation of M3R and Wnt/β-catenin signaling in order to make normal colon epithelial cells convert into CSC [78](Figure 2).

### 3.2. Trimethylamine n-oxide (TMAO) is related to CRC by unknown underlying mechanisms

Trimethylamine N – oxide is a Trimethylamine (TMA) oxidation product, belonging to the amine oxide family [79]. TMA consists of dietary choline and phospholipids (lecithin) under the effect of the human gut microbiome [80,81]. The host absorbs TMA and it is then processed by flavin-containing monooxygenases (mainly FMO3) in the liver to TMAO and excreted via the kidneys [82,83]. A prospective cohort study including 835 CRC cases and 835 matched controls discovered that increased plasma levels of TMAO are related to a greater CRC risk [84]. Another study discovered a significant association between TMAO and CRC, and noted that TMAO engaged in a number of genetic pathways with an apparent association to carcinomas, in particular colon cancer [85]. Although there is evidence suggesting elevated TMAO levels are related to an increased cancer risk, it remains uncertain whether elevated TMAO levels are a reason for or a result of cancer [86], so the role of TMAO in promoting cancer remains controversial. Currently, evidence suggests that inflammation is a potential contributor to the connection between TMAO and cancer [87], but further evidence is required in order to validate this.

### 3.3. Hydrogen sulfide (H_2_S) regulates the growth or death of cells and promotes the proliferation of CRC

H_2_S is created by *Sulfate-reducing bacteria* (SRB) metabolizing sulfates in food, and other sulfur-containing compounds, including taurine [88]. There is evidence suggesting that the level of H_2_S in CRC subjects’ feces is higher than in the control group without tumors [89]. Another study discovered significantly higher fecal H_2_S levels in patients with colon tumors and sigmoid surgery compared to healthy individuals of a similar age [90], meaning that the ability of colon detoxification H_2_S is also reduced in colon cancer patients [91]. It can therefore be suggested that H_2_S will likely work in the pathogenesis of intestinal diseases, inflammatory bowel diseases (IBDs,) and CRC [92]. Studies on the role played by H_2_S in CRC have reached different conclusions. Some studies believe that H_2_S promotes CRC due to its ability to promote inflammation and genotoxicity at physiological concentrations [93,94], in addition to being able to inhibit butyric acid oxidation and promote cell proliferation in vitro [95,96]. The pro-inflammatory effect of H_2_S appears to be related to the disruption of disulfide bonds in the double layer of mucus in the colonic wall by H_2_S, which leads to the epithelium being exposed to bacteria and toxins [97]. Interestingly, some studies suggest that H_2_S can protect the mucus layer and reconstitute the already disrupted mucus layer, thereby preventing inflammation [98,99]. Several studies have confirmed this using new non-steroidal anti-inflammatory drugs (NSAID) which release H_2_S [100,101]. H_2_S-releasing compounds exhibit potent anticancer effects through inhibition of the proliferation and/or inducing apoptosis in several types of cancer cell, including CRC, but the mechanism that is involved remains unknown and could be related to H2S inhibiting nuclear factor-κB (NF-κB) signaling and increasing intracellular Ca2+ concentration, which leads to cell cycle arrest [102,103]. Generally, the role of H2S in CRC is controversial and further study of the mechanistic pathways is required.

### 3.4. Bacterial toxins can cause DNA damage, promote inflammation, and regulate tumor microenvironment for promotion of CRC occurrence and invasion

In addition to transforming nutrients, bacteria also affect CRC by producing carcinogenic metabolites or toxic factors. These toxins can be characterized by genotoxicity, pro-inflammatory, and epithelial infiltration, and can induce and promote CRC occurrence. The commonly held belief is that there is a complex interaction between bacterial toxins and CRC occurrence.

B.fragilis toxin (BFT), which is the main virulence factor *Enterotoxigenic bacteria fragilis* (ETBF) produces, is a 20kDa zinc-dependent metalloproteinase toxin with three isotypes (BFT-1, BFTt-2, and BFT-3), all of which demonstrate similar biological activity [104]. BFT induce lysis of the extracellular domain of intercellular adhesive protein (E-cadherin) in vitro, cause a loss of epithelial integrity and increase the permeability of the single layer of colonic epithelial cells (CEC) [105], while triggering the activation of Wnt by β-catenin localization (inducing transcription and translation of the proto-oncogene c-Myc, which promotes CEC proliferation) [106]. BFT also activates NF-κB signaling, causing CEC to secrete cytokines, and potentially promoting mucosal inflammation. Enhanced NF-κB signaling can assist in CEC’s oncogenic effects [107]. Bacterially produced BFT up-regulates the spermidine oxidase (SMOX) gene that is expressed in human healthy CEC [108], and SMOX codified SMO (smooth) protein is essential for the normal metabolism of polyamines. SMO promotes the conversion of spermine to spermidine, and generates hydrogen peroxide and aldehydes, which causes DNA damage and apoptosis, and promotes cancer growth [109].

**Figure 3.**
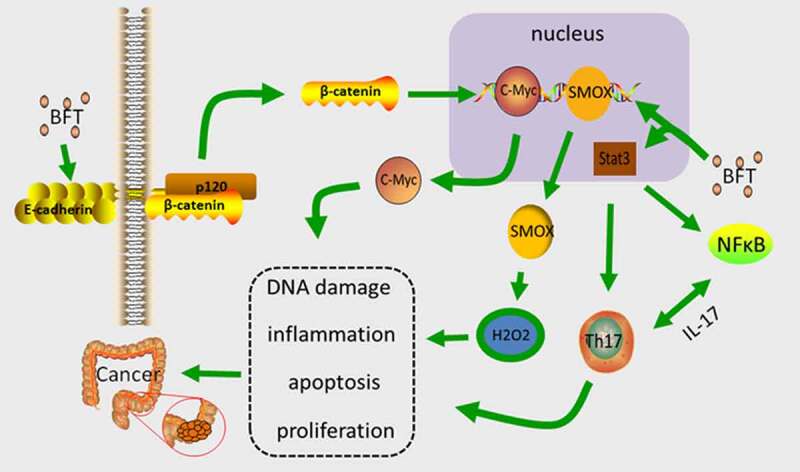
The main mechanism of BFT carcinogenesis. BFT up-regulates the expression level of SMOX and increases the synthesis of SMO, which promotes the conversion of spermine, which produces hydrogen peroxide causing DNA damage and apoptosis, and promotes the progression of cancer. BFT can induce cleavage of E-cadherin, trigger activation of Wnt by β-catenin nuclear localization, induce transcription and translation of c-Myc, and promote proliferation of CEC. BFT activates Stat3 to regulate Th17 cells, produce IL-17, and promote tumor development

Stat3(signal transducer and activator of transcription 3) is a multifunctional transcription factor in which affects the pathogenesis of autoimmunity by binding to numerous genes that are associated with Th17 cell division, activation, and multiplication, while mediating expression and epigenetic alterations [110]. BFT can activate Stat3 for the regulation of Th17 cells [111]. IL-17 is mostly produced by Th17 cells [112,113], and endogenous IL-17 is tumorigenic, which directly affects CEC signaling, cell survival, and proliferation [114]. In addition, activated NF-κB has been observed in IL-17-stimulated cells, whereas the activation of Stat3 maintains the activity of NF-κB [115]. ETBF can selectively activate Stat3 in the colon, inducing Th17 cell infiltration to cause colon cancer. After blocking IL-17, ETBF can inhibit colon tumor which is induced by ETBF [116](Figure 3).

The cell surface protein FadA is a key poison factor for *fusobacterium nucleatum* (Fn), regulating the adhesion and invasions of bacteria. The expression of FadA gene was obviously increased in CRC patient specimens in comparison to normal tissues [117]. It exists in two forms, a non-secretory pre-FadA which consists of 129 amino acid (AA) residues, and a secretory maturing FadA (mFadA) which consists of 111AA and has a signal sequence of 18 AA [118]. Intrinsic FadA and mFadA precomplexes secure Fn binding and invade host epithelial cells [119]. The binding of FadA to host endothelial receptors and vascular endothelial globulin (CDH5) is essential for Fn adhesion and cell invasion and results in the detachment of CDH5 from the cellular junction, which increases endothelial permeability and allows bacteria to pass through the loose junctions [120]. FadA can also be bound to E-cadherin in vitro. E-cadherin exists in epithelial cells, including non-cancerous HEK293, and CRC cells with the exception of RKO. FadA mediates Fn adhesion and the invasion of CRC cells through E-cadherin [117]. FadAc specifically binds to E-cadherin, which leads to phosphorylation and the internalization of E-cadherin on the membrane, thereby inhibiting its tumor suppressor activity. This results in elevated β-catenin-regulated transcription and triggers an inflammatory response, which increases the gene expression of the transcription factor NF-κB and Wnt pathways, and promotes CRC cell proliferation [121].

The main toxins that intestinal bacteria produce are colistin and CDT (cytolethal distending toxin), which are made by *Escherichia coli* (*E. coli)* and other gram-negative bacteria in the large intestine and directly damage DNA [116,122,123]. Colibactin is a heterogeneous ketone compound/non-ribosomal peptide complex that is produced by a complicated biosynthetic mechanism [124]. Certain strains which produce myxomycetin often have an association with CRC [125,126]. Colibactin can cause the breakage of double-strand DNA, chromosome instability, and cell senescence in eukaryotic cells [122,126–128]. Bacteria which produce E. coli can modify the tumor microenvironment, which leads to cellular aging and can also influence tumor progression through the secretion of growth factors [129]. CDT are bacterial protein family toxins that are produced by a number of gram-negative bacteria, including *E. coli, actinomycetes, shigella dysentery*, and *helicobacter pylori* [130]. The genotoxin CDT consists of three subunits CdtA, CdtB, and CdtC. CdtB is similar to DNAase I and can cause damage to host DNA [131]. CdtA and CdtC subunits are required proteins which mediate the combination of toxins with target cytoplasmic membranes and allow the internalization of essential active subunit CdtB [132]. CDT can trigger a DNA damage response, which leads to the arrest of the cell cycle and causes cellular senescence or death [133–135]. CDT has a critical effect on the carcinogenic effect that *campylobacter jejuni* induces. CDT-derived *campylobacter jejuni* cause injury to host cell DNA, and promote colorectal tumorigenesis by triggering cell multiplication and the enhancing of nuclear translocation of β-catenin protein [131].

## 4. Outlook and conclusion

Colorectal cancer is a multifactorial disease and microbial dysbiosis in the human gut that has been identified as a danger factor for CRC [136–138]. Although the underlying mechanisms of the role of microbial products in CRC is not fully understood, the use of dietary regulation or probiotics in CRC control has been investigated. The gnotobiotic mouse model discovered that dietary fiber and human gut microbiome can regulate the colon lumen of butyric acid salt level, and therefore the structure and colonic crypt in the presence of stromal cells, allowing the development of its role of inhibiting colon cancer in the body. These findings suggest that probiotics and/or prebiotics can be used in order to elevate the endogenous HDAC inhibitors’ content and reduce tumor development, without the adverse reactions similar to those caused by the use of synthetic HDAC(e.g., vorinostat/SAHA) in chemotherapy [139]. Gut microbiome can influence cancer chemotherapy’s efficacy and toxicity [140]. In an animal model, the co-administration of *bifidobacterium long* and *bifidobacterium short* can improve cancer control, significantly reduce tumor progression and enhance the PD-L1 blocking antibody’s anticancer effect [141]. It has also been demonstrated that gut microbiota disorders can lead to the reduced antitumor efficacy of 5-Fluorouracil (5-FU) [142]. Although these results cannot be directly applied to the treatment of human cancer, they offer the potential to use microorganisms for the prevention of CRC.

To summarize, gut microbes’ products can play an important role in the prevention of CRC (Table 1) or the promotion of CRC progression (Table 2) using various mechanisms. Limited studies have been conducted which explore the relationship between gut microbiota and CRC. Further evidence is required to support a causal relationship between human gut microbiome in CRC, and more clinical data is required to support the feasibility of microbial prevention and treatment of CRC.

**Table 1.** Factors inhibiting CRC

Origins	Products		Potential microbe	Mechanism	References
dietary fibers and starches	SCFA	Butyrate	*ruminococcaceae* *lachnospiraceae*	Function as HDAC inhibitor	20,23
				Down-regulate miR-92a expression via c-Myc	29
				Upregulates miR-203	34
		Acetate	*Propionibacterium*	DNA fragmentation	39,40
				Caspase-3 activation	39,40
		Propionates	*Propionibacterium*	Induces PRMT1 downregulation	41
BA	UDCA		*Clostridium absonum, Clostridium baratii,*	Decreasing the concentration of hydrophobic BA	51
				Regulate oxidative stress	52
				Up-regulates colonic MHC expression, enhancing immune surveillance	53
				Inhibits NF-κB activated	54
Unknown	Niacin		*Lactobacillus acidophilus*	Enhances anti-inflammatory effects by activating GPR109a	59
				Effect on CSC	63
Sulfates and other sulfur-containing compounds	H2S		*Sulfate-reducing bacteria*	Inhibit NF-κB signaling and increase intracellular Ca2+ concentration, leading to cell cycle arrest	101,102

CRC: colorectal cancer; SCFAs: short chain fatty acids

HDAC: histone deacetylase;

MiR-92a: microRNA-92a;

BA: bile acids

UDCA: ursodeoxycholic acid

MHC: major histocompatibility

NF-κB: NF-kappaB

GPR: G protein-coupled receptors

CSCs: cancer stem cells

H2S: Hydrogen sulfide

**Table 2.** Factors promoting CRC

Origins	Products	Potential microbe	Mechanism	References
BA	DCA and LCA	*Clostridium, Enterococcus, Bifidobacterium, Lactobacillus*	produce ROS and RNS, causing oxidative stress, and damaging DNA	69
			K-ras point mutual mutations	70
			Stimulate EGFR-MARK signaling	70
			Regulate M3R and Wnt/β-catenin signaling	78
Choline and phospholipids	TMAO	Unknown	Promotes inflammation	87
Sulfates and other sulfur-containing compounds	H2S	*Sulfate-reducing bacteria*	Promotes inflammation, genotoxicity, inhibits butyric acid oxidation	93–96
Bacterial toxins	B.fragilis toxin (BFT)	*Enterotoxigenic bacteria fragilis* (ETBF)	Induces E-cadherin lysis, resulting in loss of epithelial integrity	105
			Triggers activation of Wnt, induces c-Myc expression and promotes CEC proliferation	106
			Activates NF-κB signaling and promotes inflammation	107
			Upregulates the expression of SMOX genes, leading to DNA damage	108,109
			Activates Stat3 to regulate Th17 cells and maintain NF-κB activity	112,116
	FadA	*Fusobacterium nucleatum* (Fn)	Binds to CDH5, increases endothelial permeability, and allows bacteria to pass through loose junctions	117
			Binds to E-cadherin and mediates Fn adhesion and invasion of CRC cells	117
			Binding to E-cadherin inhibits its tumor suppressor activity and increases the expression of NF-κB and Wnt pathways	121
	Colibactin	E. coli and other gram-negative bacteria	Leads to double-stranded DNA breaks, chromosomal instability and cellular senescence	122,126–128
	Cytolethal distending toxin (CDT)		Causes DNA damage, leading to cell cycle arrest and cellular senescence or death	133–135

CRC: colorectal cancer

BA: bile acids

DCA: deoxycholic acid

LCA: lithocholic acid

ROS: reactive oxygen species

RNS: reactive nitrogen substances

EGFR: epidermal growth factor receptor

MAPK: mitogen activated protein kinase

TMAO: trimethylamine n-oxide

H2S: Hydrogen sulfide

BFT: fragile bacteroides toxin

ETBF: enterotoxigenic fragile bacteroides

CEC: colonic epithelial cells

SMOX: spermine oxidase

NF-κB: NF-kappaB

Fn: Fusobacterium nucleatum

CDT: cytolethal distending toxin
